# Effects of blended learning training for oncology physicians to advise their patients about complementary and integrative therapies: results from the multicenter cluster-randomized KOKON-KTO trial

**DOI:** 10.1186/s12885-023-11348-6

**Published:** 2023-09-07

**Authors:** Stefanie M. Helmer, Alizé A. Rogge, Ryan King, Claudia Canella, Daniel Pach, Claudia M. Witt

**Affiliations:** 1 Institute of Health and Nursing Science, Charité – Universitätsmedizin Berlin, corporate member of Freie Universität Berlin and Humboldt-Universität zu Berlin, Berlin, Germany; 2 Institute of Public Health and Nursing Research, Faculty 11 Human and Health Sciences, University of Bremen, Bremen, Germany; 3 Department of Psychosomatic Medicine, Center for Internal Medicine and Dermatology, Charité - Universitätsmedizin Berlin, corporate member of Freie Universität Berlin and Humboldt-Universität zu Berlin, Berlin, Germany; 4 Institute of Social Medicine, Epidemiology, and Health Economics, Charité – Universitätsmedizin Berlin, corporate member of Freie Universität Berlin and Humboldt-Universität zu Berlin, Berlin, Germany; 5 Institute for Complementary and Integrative Medicine, University Hospital Zurich and University Zurich, Sonneggstrasse 6, 8091 Zurich, Switzerland

**Keywords:** Integrative oncology, Cancer, Physician–patient communication, Complementary medicine, Clinical trials

## Abstract

**Background:**

Many oncology physicians are confronted with the topic of complementary and integrative medicine (CIM) by cancer patients. This study examined whether a blended learning (e-learning and a workshop) to train oncology physicians in providing advice on CIM therapies to their cancer patients, in addition to distributing an information leaflet about reputable CIM websites, had different effects on physician-reported outcomes in regard to consultations compared with only distributing the leaflet.

**Methods:**

In a multicenter, cluster-randomized trial, 48 oncology physicians were randomly allocated to an intervention group (CIM consultation and an information leaflet) or a control group (information leaflet only). After the training, the oncology physicians conducted 297 consultations with their cancer patients. Measurements were assessed at oncology physician, physician–patient-interaction (measured by external reviewers), and patient levels. This analysis focused on the physician outcomes of stress reaction and perceived consultation skill competency. In addition, qualitative interviews were conducted with a subsample of oncology physicians who experienced both, the intervention and control condition.

**Results:**

The oncology physicians in the intervention group showed a lower stress reaction in all measured dimensions after CIM consultations than those in the control group. There was no significant difference between oncology physicians in the intervention and control groups regarding the perceived consultation skill competency (overburden: intervention 1.4 [95% CI: 0.7;2.1]; control 2.1 [95% CI: 1.4;2.7], tension: 1.3 [95% CI: 0.7;2.0] vs. 1.9 [95% CI: 1.3;2.5], and discomfort with consultation situations: 1.0 [95% CI: 0.4;1.7]; vs. 1.7 [95% CI: 1.2;2.3]). The qualitative data showed that only providing the leaflet seemed impersonal to oncology physicians, while the training made them feel well prepared to conduct a full conversation about CIM and provide the information leaflet.

**Conclusions:**

In our exploratory study providing structured CIM consultations showed positive effects on the perceived stress of oncology physicians, and the training was subjectively experienced as an approach that improved physician preparation for advising cancer patients about CIM, however no effects regarding perceived consultation skill competency were found.

**Trial registration:**

The trial registration number of the KOKON-KTO study is DRKS00012704 in the German Clinical Trials Register (Date of registration: 28.08.2017).

**Supplementary Information:**

The online version contains supplementary material available at 10.1186/s12885-023-11348-6.

## Background

Many oncology physicians are confronted with the topic of complementary and integrative medicine (CIM) in their daily work. Many cancer patients already use CIM or ask their oncology physicians about CIM therapies [1–3]. Good communication about CIM between oncology physicians and their cancer patients could help to reduce potential pharmacological interactions between CIM and cancer treatment [4]. Furthermore, good communication can lead to higher levels of trust in cancer treatment and compliance, as well as increased patient satisfaction, less stress, and greater well-being [5]. To overcome knowledge- and communication-based challenges, structured training in the use of CIM in oncology is urgently required by oncology physicians [6, 7].

Generally, communication skills training for health care professionals who work with cancer patients has been widely implemented in recent years. Many of these trainings lead to improved communication skills, and oncology physicians who participate in such trainings are less likely to provide facts alone without individualizing their responses to the patient’s emotions [8]. However, evidence is limited as to whether training oncology physicians is not only beneficial for oncology physicians themselves but also well-tailored to the counseling needs of patients [8]. A paper from a consensus meeting among European experts emphasizes that trainings should support oncology physicians in feeling confident in mastering difficult communicative tasks. Moreover, it was agreed that training should enable physicians to respectfully take their patient’s clinical and personal context into account. In terms of educational methods, interactive and experiential approaches such as feedback and role-play are recommended so that physicians can reflect on themselves, their learning progress, and their communication [9].

An interprofessional consensus procedure focusing on competencies that are of specific interest for trainings in integrative oncology found that such consultations are accompanied by unique challenges [10]. For instance, there are many potential complementary and integrative medicine options with highly variable evidence bases [11], and a CIM consultation thus requires not only fundamental knowledge of CIM therapies but also the ability to provide evidence-based, balanced, resource-oriented, up-to-date CIM information that assists patients in making decisions. Moreover, oncology physicians should be open-minded and take patients’ beliefs into account [10].

In addition to the competencies taught, it is important that the information about CIM that is necessary to inform a patient is tailored to the needs of the physicians and to the consultation situation. Oncology physicians prefer lectures, face-to-face workshops, and flexible online education that is accessible all the time [12]. In regard to the consultation situation, the experience of a previously conducted trial shows that CIM consultations cannot be easily integrated into everyday practice due to time constraints and should therefore be brief and adjusted to the needs of both parties in the interaction [13].

The KOKON-KTO study was part of the Competence Network Complementary Medicine in Oncology (KOKON), a collaborative research project in Germany that was funded in a second funding period by German Cancer Aid [14]. The KOKON-KTO study (KTO = Consultation Training for Oncology Physicians) aimed to evaluate whether training oncology physicians to advise their cancer patients on CIM, in addition to distributing an information leaflet about reputable websites, had different effects on outcomes at the patient, oncology physician, and physician–patient levels compared to only distributing the information leaflet. The evidence-based blended learning training is tailored to oncology physicians with little experience in CIM who advise cancer patients about CIM in only 20 min [15]. This paper focuses mostly on oncology physician-reported outcomes.

## Methods

### Design

The KOKON-KTO study was a prospective, multicenter, cluster-randomized trial in which oncology physicians trained in giving CIM advice (intervention) were compared to an untrained group of the same profession (control). Following the quantitative study, qualitative data were also assessed to learn more about the consultation from oncology physicians who could relate to both consultation settings. A detailed description of the study design can be found elsewhere [15].

### Sample

A total of 48 oncology physicians (50% who specialize in gynecologic oncology) from hospital departments or private practices in Germany were included in the study. All potential participants needed to have a specialization in the field of oncology and were screened in advance in a telephone interview conducted by one of the authors. If they answered the following questions with yes, they were included: little knowledge of CIM, no previous structured trainings in the use of CIM in the field of oncology, little experience in advising cancer patients on CIM, sufficient resources to conduct consultations with cancer patients. Little knowledge of CIM was operationalized as exceeding basic knowledge that was taught during standard medical training. Little experience in advising cancer patients on CIM was operationalized as actively providing information on CIM to oncology patients. In addition, it was required that oncology physicians have sufficient resources to conduct consultations with cancer patients, were able to attend the workshop, and have good German language skills. After completing the training (intervention or delayed intervention for control oncology physicians), the participants received 34 Continuing Medical Education (CME) points from the German Doctors Association.

All the participating oncology physicians completed written informed consent forms and were told that they could withdraw from the intervention at any time during the study.

Hospital departments or private practices served as clusters. From each hospital department, two oncologists had to be eligible and served as one cluster. Randomization of clusters was stratified by type of center (hospital department or practice) and specialization (gynecology, other type of oncology) using an allocation ratio of 1:1. The randomization was performed by personal not otherwise involved in implementation of the study or analysis. After randomization, one oncology physician specializing in gynecologic oncology working in a hospital department was not able to participate in the study for personal reasons.

### Intervention

The training framework was previously published [16]. In brief, oncology physicians in the intervention group took part in a two-phase intervention. First, they participated in a blended learning (e-learning and workshops) that qualified oncology physicians to provide structured advice to their cancer patients on CIM using the KOKON-KTO consultation manual. The KOKON-KTO consultation manual was developed specifically for this occasion based on educational competencies [10] and evidence-based CIM knowledge [16]. Second, each intervention physician conducted consultations following the KOKON-KTO consultation manual with their own patients and provided an information leaflet about reputable websites on the use of CIM in oncology (KOKON-KTO information leaflet). Oncology physicians in the control group participated in a three-phase control intervention. First, the oncology physicians participated in a short e-learning that introduced them to the study and the KOKON-KTO information leaflet. Second, the oncology physicians in the control group provided the KOKON-KTO information leaflet to their own patients along with a short introduction to the leaflet and its use. Third, the oncology physicians in the control group also received KOKON-KTO training.

### Data assessment

All oncology physicians were asked to answer questionnaires at multiple time points during the KOKON-KTO study. First, a web-based survey prior to e-learning was implemented (baseline MD). Second, paper–pencil questionnaires were distributed to the oncology physicians to provide information after each communication with their patients or provision of the information leaflet to their patients, respectively. Third, each oncology physician received a paper–pencil questionnaire after having conversations with as many patients as they were able to enroll in the study. For the physician–patient interaction level, a systematic external rating of a consultation of the participating oncology physicians was conducted by two experienced independent raters. For this, a consultation with a standardized patient was undertaken during the onsite skills-training workshop in the intervention and control groups.

### Outcomes

Outcome measures for the evaluation of training in oncology have been defined based on an international consensus process [17]. Within this process, international experts discussed possible outcomes that have been identified as the most important outcomes within a systematic literature review. Workshop experts suggested that several outcomes should be assessed on three different levels: outcomes related to (1) the oncology physicians, (2) the specific interaction between oncology physicians and patients, and (3) patients who communicate with oncology physicians [17].

- a) Physician level.

Two main outcomes at the physician level were selected: (1) the perceived consultation skill competency, indicated by three questions on subjective experiences of overburden, tension, and discomfort with the consultation situation; and (2) the perceived stress reaction, indicated by feeling secure at various moments in a CIM consultation (five questions on assessing previous CIM experiences, giving information about the effectiveness of CIM therapies, dealing with questions about unclear evidence, staying informed about interactions between antitumor therapy and CIM, and giving concrete advice). Both instruments were self-developed instruments as they needed to be tailored to the specific KOKON-KTO consultation setting and were measured on numerical rating scales (NRS) ranging from not at all (0) to fully competent/secure (10). The oncology physicians were asked to complete the perceived consultation skill competency instrument after each consultation, and the perceived stress instrument after all conducted consultations.

Further outcomes were the level of CIM knowledge in cancer care (multiple-choice questions); expectations regarding the effectiveness of CIM (5-point Likert scale from fully agree (5) to fully disagree (1)) and expectations regarding the side effects of CIM (NRS from very safe (0) to not safe at all (10)). Moreover, the oncology physicians were asked to indicate the duration of the CIM consultation in minutes. If the consultation took more than the anticipated 20 min, the oncology physicians were asked to provide reasons. All these questions were asked at baseline and after the training.

Furthermore, to obtain more information about the feasibility of the intervention, the physicians were asked questions after conducting all conversations. The ability of the manual-based consultation to be implemented in the treating oncology physicians’ daily work was measured on a 6-point Likert scale from very good (1) to not at all (6). Furthermore, the oncology physicians were asked to indicate their attitude toward CIM on four levels (the importance of being well informed about CIM, feeling confident during conversations, trying to avoid CIM conversations with patients, wishing that the patient would engage less in CIM) using an NRS from fully disagree (0) to fully agree (10)).

- b) Patient level.

To learn more about the usefulness of the information leaflet about reputable websites on the use of CIM in oncology (KOKON-KTO information leaflet) that all the oncology physicians provided to their patients, the patients were asked whether they used the recommended websites. The response options were: No; Not yet, but I want to use it in the future; and Yes.

- c) Physician–patient interaction level.

As a surrogate variable for indicating the physician–patient interaction, oncology physicians in the intervention group were asked to lead one KOKON-KTO consultation with a standardized cancer patient during the KOKON-KTO workshop. During these consultations, two independent external raters observed whether the oncology physicians followed the structure of the KOKON-KTO consultation [16] and rated the interactive and communicative competencies that are necessary for a good physician–patient interaction. The interactive and communicative competencies were rated using the “Munich Physician Patient Interaction Inventory” (MAPI) [18] (11 questions; 5-point Likert scale). All the raters were trained for reliability. For interactive and communicative competencies, the interrater reliability was substantial (average interclass correlation, R^2^ = 0.77 ± 0.19).

### Statistical analyses

Mean values and standard deviations were calculated for metric variables, and frequencies and percentages were calculated for categorical variables. To investigate the effects of the intervention on those outcome variables assessed after each consultation, linear mixed models (LMMs) were employed in an intent-to-treat analysis. Fixed effects were the control group effect, the intervention group effect, and the variables with which the randomization was stratified (hospital/practice and oncology/gynecology). The clustering of patients treated by one physician was included as a random effect. Linear regression was performed as intent-to-treat for variables assessed once after conducting all consultations, and was adjusted for baseline, hospital/practice and oncology/gynecology. As suggested in the review process an additional analysis was performed reflecting the randomization by center; using the individual centers as clusters (random effect) instead of the single physician within each center (see Supplemental Materials). Since all statistical analyses are considered exploratory, adjusted group means and 95% confidence intervals are displayed, and no sample size was calculated.

Sensitivity analyses were performed for the per-protocol population (oncology physicians having KOKON-KTO consultations with at least five patients). Additionally, we controlled for the sex of the oncology physicians. Statistical analyses were conducted using RStudio [19].

### Qualitative data

Qualitative data were collected in phase III of the study. Oncology physicians in the control group received KOKON-KTO training following the intervention phase as an incentive to participate in the study [15]. Furthermore, these oncology physicians were asked to voluntarily conduct the KOKON-KTO consultation with up to five of their cancer patients. Since this group of oncology physicians was experienced in both settings (conducting the KOKON-KTO consultation, and in short consultations only providing the KOKON-KTO information leaflet), semistructured telephone interviews were conducted 8–10 weeks after the KOKON-KTO training to asses both settings. The physicians were asked about: 1) their positive or negative experiences with both settings and possible reasons for these; 2) their own and their perception of the patients’ satisfaction with both settings; and 3) possible differences between the two settings (see Rogge et al. [20] for further information on the interview guidelines).

After nine interviews, an acceptable saturated spectrum of expressed topics and experiences [21] was reached. The interviews for the qualitative part were audiotaped and transcribed verbatim. A qualitative content analysis, following the methods described by Flick [22] and using the qualitative data analysis software MAXQDA [21], was performed. The transcripts were coded in sense units combining deductive and inductive coding strategies. The research team predefined deductive codes according to the KOKON-KTO consultation manual [16] and added further subcategories in a continuous process of inductively building codes from the data, and an intersubjective validation of the coding by two independent researchers (AAR and CC) to verify the reliability and robustness of the data analysis.

## Results

### Baseline characteristics of oncology physicians and study flow

A total of 47 oncology physicians (*n* = 23 in the intervention group, *n* = 24 in the control group) completed the questionnaire (baseline MD) at baseline. Twenty-one oncology physicians in the intervention group completed the e-learning modules of the KOKON-KTO training, and 2 did not continue with the study due to changes of workplace or personal reasons. After the e-learning session, 1 oncology physician in the intervention group could no longer participate in the workshop for health reasons.

All the oncology physicians were asked to conduct up to 10 KOKON-KTO conversations each with their cancer patients and to provide the KOKON-KTO information leaflet (intervention group) or only provide the leaflet to patients (control group); then, the physicians were asked to complete a questionnaire after each consultation/provision of the KOKON-KTO information leaflet (t_1_ MD). The average number of consultations per oncology physician was 5.6 in the intervention group and 7.0 in the control group. Two oncology physicians in the intervention group were not able to enroll any patients in the study due to a lack of suitable cancer patients in their hospital. After completing all consultations with their cancer patients (t_2_ MD), the follow-up questionnaire was answered by 18 oncology physicians in the intervention group and 23 oncology physicians in the control group. A detailed description of the study flow can be found elsewhere [20].

The demographic characteristics of the participating oncology physicians are presented in Table 1. In total, 70.2% of oncology physicians were female (intervention group: 60.9%, control group: 79.1%). The oncology physicians in the intervention group were slightly younger and had fewer years of experience working with patients.

**Table 1:** Oncology physicians’ characteristics

	Intervention (*n* = 23) mean (± sd)/n (%)	Control (*n* = 24) mean (± sd)/n (%)	Total sample (*n* = 47) mean (± sd)/n (%)
Sex, female (%)	14 (60.9)	19 (79.2)	33 (70.2)
Age in years, mean (SD)	40.5 (± 8.9)	42.0 (± 10.6)	41.3 (± 9.7)
Level of occupational qualification (%)			
Hospital department physician in further training	9 (39.1)	9 (37.5)	18 (38.3)
Hospital department specialist physician	2 (8.7)	3 (12.5)	5 (10.6)
Hospital department senior or chief physician	5 (21.7)	5 (20.8)	10 (21.3)
Physician in private practice	7 (30.4)	7 (29.2)	14 (29.8)
Number of years of experience with cancer patients, mean (SD)	11.0 (± 7.6)	13.3 (± 10.7)	12.2 (± 9.2)
Included patients in study	128	169	297
Number of KOKON-KTO conversations with patients, mean (SD)	5.6 (± 4.6)	7.0 (± 3.7)	6.3 (± 4.2)

Of the 297 included patients, 84.2% were female and 55.0 years old on average (intervention: 54.1 ± 10.6; control: 55.6 ± 11.8). The graduation level of 120 patients was A-Level or higher (intervention: 49 (30.4%) vs. control: 71 (42.0%)). Fifty-six percent of patients were diagnosed with breast cancer (intervention: 60.9% vs. control: 52.1%) and 9.8% with other gynecological cancers (intervention: 7.8% vs. control: 11.2%). Oncology physicians in the control group treated patients with more severe tumors, such as gastrointestinal tumors (intervention: 10.2% vs. control: 15.4%) and pulmonological tumors (intervention: 3.9% vs. control: 7.1%). The treatment objective of 62.3% of patients (intervention: 68.8% vs. control: 57.4%) was curative/adjuvant, for 34.3% it was palliative (intervention: 26.6% vs. control: 40.2%), and for 3.4% (intervention: 5.7% vs. control: 2.3%) it was not clear.

- a) Physician level.

In regard to the anticipated effectiveness of specific CIM therapies, physical activity during anti-tumor therapy was concordantly rated as effective among most intervention oncology physicians, followed by acupuncture (95.2% agreement). More oncology physicians rated mistletoe, ginseng, and acupuncture as effective following e-learning, whereas a substantial decrease was found for the rated effectiveness of curcuma. Mindfulness training and acupuncture were considered safe CIM therapies by most oncology physicians after the training. The percentage of participants who considered hypnosis, mistletoe, and acupuncture safe increased, and the percentage of participants who considered physical activity safe decreased. The results of both time points are presented in Table 2.

**Table 2:** Oncology physicians’ ratings of their anticipated CIM effectiveness (in %) and CIM safety

	**Effectiveness: high (fully agree)** n (%)		**Safety: high (7–10 Categories)**^**a**^ n (%)	
	Rating at baseline (*n* = 23)	Rating after e-learning (*n* = 21)	Rating at baseline (*n* = 23)	Rating after e-learning (*n* = 21)
**Acupuncture**	18 (78.3)	20 (95.2)	17 (73.9)	20 (95.2)
**Homeopathy**	4 (17.4)	5 (23.8)	14 (60.9)	14 (66.7)
**Yoga**	18 (78.3)	18 (85.7)	21 (91.3)	19 (90.5)
**Nutrition during antitumor therapy**	21 (91.3)	18 (85.7)	18 (78.3)	19 (90.5)
**Physical activity during antitumor therapy**	22 (95.7)	21 (100)	22 (95.7)	19 (90.5)
**Fasting**	6 (26.1)	8 (38.1)	5 (21.7)	8 (38.1)
**Mindfulness training**	16 (69.6)	18 (85.7)	19 (82.6)	20 (95.2)
**Hypnosis**	5 (21.7)	7 (33.3)	7 (30.4)	14 (66.7)
**Ginseng**	1 (4.3)	5 (23.8)	5 (21.7)	8 (38.1)
**Devil’s Claw (** ***Harpagophytum procumbens*** **)**	2 (8.7)	3 (14.3)	5 (21.7)	4 (19.0)
**Cimicifuga**	4 (17.4)	7 (33.3)	4 (17.4)	6 (28.6)
**Curcuma**	5 (21.7)	2 (9.5)	7 (30.4)	7 (33.3)
**Vitamin C**	5 (21.7)	6 (28.6)	6 (26.1)	8 (38.1)
**Medical Mushrooms**	1 (4.3)	3 (14.3)	2 (8.7)	5 (23.8)
**Mistletoe**	8 (34.8)	14 (66.7)	6 (26.1)	13 (61.9)

^a^Numeric Rating Scale 0 (very unsafe)-10 (very safe)

The duration of consultations in the intervention group was 22.6 min on average (SD = 4.7). The oncology physicians in the control group communicated with their cancer patients for an average of 7.1 min (SD = 4.2).

Regarding the perceived stress reaction, oncology physicians in the intervention group scored lower than those in the control group on all five items. There was no significant difference between oncology physicians in the intervention and control group regarding perceived consultation skill competency. However, the mean scores were lower for all three levels of consultation skills (overburden: intervention 1.4 (95% CI: 0.7;2.1), control 2.1 (95% CI: 1.4;2.7); tension: intervention 1.3 (95% CI: 0.7;2.0), control 1.9 (1.3;2.5); and discomfort with the consultation situation: intervention 1.0 (95% CI: 0.4;1.7), control 1.7 (95% CI 1.2;2.3)) (Table 3).

**Table 3:** Primary outcomes at the physician level in the intention-to-treat sample. Linear Mixed Model analyses (adjusted means and differences between the intervention and control group)

	**Intervention group (** ***n*** **=18) ** **mean (95% CI)**	**Control group (** ***n*** **=23) ** **mean (95% CI) **	**Group differences** mean (95% CI)	***p*** ** value **
**Perceived stress reaction **				
assessing previous CIM	8.4 (7.4;9.4)	5.8 (4.9;6.7)	2.6 (1.3;3.9)	<0.001
providing information about effectiveness of CIM therapies	7.8 (6.7;8.8)	3.8 (2.9;4.8)	4.0 (2.5;5.4)	<0.001
addressing with questions about unclear evidence	7.3 (6.2;8.5)	3.7 (2.6;4.7)	3.7 (2.1;5.2)	<0.001
staying informed about interactions between anti-tumor therapy and CIM	7.8 (6.7;8.8)	3.6 (2.6;4.7)	4.1 (2.6;5.6)	<0.001
providing concrete advice	7.7(6.7;8.8)	3.4(2.4;4.4)	4.4 (2.9;5.8)	<0.001
**Perceived consultation skill competency **				
Overburden	1.4 (0.7;2.1)	2.1 (1.4;2.7)	-0.6 (-1.6;0.3)	0.168
Tension	1.3 (0.7;2.0)	1.9 (1.3;2.5)	-0.6 (-1.5;0.3)	0.184
Discomfort with the consultation situation	1.0 (0.4;1.7)	1.7 (1.2;2.3)	-0.7 (-1.6;0.1)	0.095

The results of the sensitivity analyses were comparable to those utilizing the intention-to-treat sample.

There was no statistically significant difference in the rating of the ability of the consultation to be implemented between the two groups, with only small differences between the groups (intervention: 5.0 (95% CI: 4.8; 5.3), control 5.2 (95% CI: 5.0;5.5)).

Regarding the attitudes of oncology physicians toward CIM, the intervention group scored statistically significantly higher in being confident in conducting conversations, and statistically significantly lower in trying to avoid CIM conversations (Table 4).

**Table 4:** Results of linear regression analyses on attitudes toward CIM

	**Intervention group (** ***n*** ** = 18)** **mean (95% CI)**		**Control group (** ***n*** ** = 23)** **mean (95% CI)**	**Differences IG-CG** **mean (95% CI)**	*p* value
Personal attitude toward CIM					
Importance of being well informed about CIM		9.0 (8.0;10.0)	7.8 (6.9;8.7)	1.2 (-0.1;2.5)	0.066
Feeling confident in conversations about CIM		7.7 (6.9;8.5)	3.4 (2.7;4.1)	4.3 (3.2;5.4)	< 0.001
Trying to avoid CIM conversations with patients		0.9 (0.0;1.7)	3.5 (2.7;4.3)	-2.6 (-3.8;-1.4)	< 0.001
Wishing that the patient would engage less in CIM		1.5 (0.6;2.4)	2.0 (1.2;2.8)	-0.5 (-1.7;0.7)	0.419

Reasons stated by participating oncology physicians in the intervention group for conducting a KOKON-KTO consultation longer than 20 min included motivating the patient to change a behavior and having an overall more detailed consultation.

- b) Patient level.

Of the 275 participating patients, the vast majority of both groups read the information leaflet about recommended CIM websites (intervention: 63.9%; control: 73.2%), and 68% of patients reported using at least one of the recommended websites. A smaller group (intervention: 20.5%; control: 15.7%) did not read the information leaflet before completing the questionnaire but were planning to do so in the future.

- c) Physician–patient interaction.

The oncology physicians who participated in the workshop were able to demonstrate interactive and communicative competencies that are necessary for good physician–patient interactions (see table in the supplemental materials). In an earlier publication was reported that oncology physicians in the intervention group were able to apply the skills they had learned during the KOKON-KTO training after the workshop [16].

### Qualitative results

Nine oncology physicians in the control group who received delayed KOKON-KTO training (88.9% were female and 42.0 ± 12.7 years old on average, 88.9% from hospital departments) reported that from the perspective of the physician leading the consultation, they felt competent and well prepared for both settings (KOKON-KTO consultation vs. only providing the KOKON-KTO information leaflet) [20]. Even though the oncology physicians still considered themselves to be lacking CIM knowledge, they found it helpful to be able to give advice on reputable online sources for CIM knowledge based on the leaflet. More than half of the oncology physicians (*n* = 6) stated that they felt as if they outsourced the topic of CIM and hence the patients’ concerns. For some oncology physicians, only providing the KOKON-KTO information leaflet felt impersonal. Moreover, some oncology physicians described doubting all patients’ abilities to use the internet. In the KOKON-KTO consultation setting, the oncology physicians felt competent and able to “meet their own quality requirements”. They liked the structure of the consultation and being an active part of it. However, some oncology physicians stated that such consultations might exceed the time frame and may “open Pandora’s box” regarding discussion of other CIM therapies that lack evidence (see the supplemental materials for the qualitative results).

## Discussion

Oncology physicians in the KOKON-KTO training group reported significantly less perceived stress after all consultations compared to the control group who only provided the KOKON-KTO information leaflet to their cancer patients. Regarding the perceived consultation skill competency measured after each consultation, no significant differences were found; however, oncology physicians receiving KOKON-KTO training scored lower on all items (less overburden, tension, and discomfort with the consultation situation) than control physicians. Oncology physicians in the intervention group were more confident in conversations and less likely to avoid CIM conversations. According to the qualitative data, the oncology physicians felt competent and well prepared for a conversation on CIM and for only providing the information leaflet, even though the latter felt somewhat impersonal.

The self-reported quantitative data of the patients in our study that have been published elsewhere support these findings. Patients who received the KOKON-KTO consultation exhibited higher levels of satisfaction in the group [20]. Both outcome levels (patient and physician) showed concordant trends between the groups, which result in a convincing basis for the KOKON-KTO training.

In a previous pilot study by Bloedt et al. [13], oncology physicians who were trained in a complementary communication blended learning program in providing advice during CIM-designated consultations achieved high patient satisfaction. However, the control group experienced comparable results, which might be explained by ceiling effects that are a common challenge in measuring the effects of communication trainings in cancer care [23, 24]. However, in this study, the view of oncology physicians who reported that additional consultations about CIM were difficult to integrate into everyday practice was also taken into account [13]. In our recent study, there was no difference in the subjective ability of the approach to be implemented between groups; therefore, 20 min appears not to interfere with everyday work.

In a study by Hayward et al., a complementary medicine practice guideline also led to positive results for health care providers, including oncologists (improvement in self-reported CIM knowledge and readiness to answer cancer patients’ questions about CIM) [25]. This education session was also created to accommodate the schedules of health care professionals and was available in multiple formats (in person as well as online). Shorter educational programs can serve as a sufficient alternative when oncology physicians aim to improve their CIM knowledge.

The oncology physicians in the control group in our study also received a short intervention and were introduced to providing an information leaflet about reputable websites on CIM. The majority of patients used the recommended websites, and the qualitative results showed that the oncology physicians found it helpful to use these resources. Sharing information leaflets might be a suitable alternative that is easy to implement in everyday care for oncology physicians who want to give their patients a helpful introduction to CIM, but also want to have more time for direct conversations on other topics [26]. The internet is a source that is frequently used to obtain information by cancer patients, but a study found that only approximately 1/3 of the obtained information influenced patients’ treatment decisions [27]. One reason for this discrepancy may be that online information is not evaluated in terms of quality and patients do not trust the information provided. Consequently, a pre-selection of high-quality, evidence-based, online resources about CIM and CIM-related adverse effects by health care providers might be helpful for cancer patients [28]. However, in addition to online information, oncology physicians should have knowledge and skills to inform themselves about specific information, such as the side effects of therapies, which could cause fear [29].

In our study, we used a blended learning method to train physicians. A review showed that it remains uncertain whether e‐learning alone improves or reduces health professionals’ skills [30]. A blended learning approach combines the advantages of online knowledge transfer, which can be adapted in its pace to physicians’ work lives with the possibility of direct interactions, exchanges, and relations with other learners, and the physical presence of the teacher during the onsite workshop.

In our study, we also measured how attitudes about the effectiveness and safety of CIM therapies changed after completing the e-learning. Oncology physicians displayed changes in the rated effectiveness and safety of most CIM therapies after participating e-learning. However, CIM therapies were rated as being effective and safe by most oncology physicians prior to the e-learning. Nutrition, physical activity, yoga, and acupuncture are the therapies that were mostly rated as effective and safe after e-learning. Patients’ expectations of CIM play a role in the treatment outcome [31]. However, to our knowledge, no studies have assessed how attitudes toward the effectiveness and safety of CIM therapies shape the recommendations of oncology physicians. Studying these associations between expectancies and attitudes toward CIM among physicians, and recommending those to patients, could help reduce bias and improve the quality of medical decision-making. For this, future research based on validated instruments as established for patient populations [32] is needed.

To our knowledge, this is the first study using a standardized framework [16], which was evaluated in a cluster-randomized trial of oncology physicians, interactions between oncology physicians and patients, and at the patient level [20]. The use of multiple assessment methods combining quantitative and qualitative measures provides a more complete evaluation of the training. However, our study has several limitations that need to be acknowledged.

Our study needs to be considered exploratory, and we did not measure effects on a single primary outcome. We involved a structured consensus approach for finding suitable outcomes for measuring effects [17], which led to the recommendation to measure several outcomes on three levels. The instruments for measuring our main outcomes were self-developed, as both need to be tailored to the specific study situation. Therefore, we do not have comparable data and no further information on the validity of the instruments. There might be over- or underestimations in specific groups due to self-reported data. We cannot rule out that oncology physicians rated their performance/the conversations more positively based on their perception in the situation. We were not able to completely blind the participants since they received a full intervention; however, the control physicians also received a short e-learning and material for their conversations (KOKON-KTO information leaflet). In addition, we used short-term (information about each consultation) and long-term (impressions after all conversations) information, which are both subject to bias. Long-term information can lead to recall bias, and oncology physicians might remember the most recent conversations more precisely. However, short-term information can be influenced by subjective emotions and might be of limited predictive value for long-term behavior. Furthermore, not all oncology physicians were able to conduct ten consultations with cancer patients, which results in limited comparability. In addition, we were not able to fully assess the implementation of the KOKON-KTO manual outside of the training situation. In our preplanned statistical analysis, we did not use the cluster as a random effect, as commonly done in cluster-randomized trials. As mentioned in our protocol publication [15]: the multilevel structure of the data is due to patients treated within a physician, and physicians working within a cluster. Because of the small number of physicians per cluster (either one or two) and the assumption that the communication varies more between single physicians than between centers, the center as cluster was not considered in the model. The results of the additional analysis, which adjusts for the specific center of the physician instead of just for the physician, did not differ in any clinically relevant way, indicating an overall robustness of the study results with respect to single physician versus center adjustment.

We did not video record the consultations based on experiences from a previous study [13]. Since the KOKON-KTO training follows a blended learning approach focusing not only on education on CIM-related knowledge but also on communication skills for oncology physicians, we cannot distinguish between components. However, we assume that both components are essential for effective communication with patients and cannot stand alone in training [5]. Moreover, even though randomization of oncology physicians took place, there were differences in the patient sample that oncology physicians selected for the conversations between the intervention and control groups. In the intervention group, there was a higher number of patients with more severe cancers, and the treatment was more frequently palliative in the control group than in the intervention group. This probably led to unequal preconditions for the conversations and may also influence stress during the conversation and one’s feeling of competence.

## Conclusion

This exploratory study evaluated a novel education intervention for training oncology physicians in providing CIM advice in routine care. Providing structured CIM consultations following a blended learning training had positive effects on the perceived stress of oncology physicians but no significant effects on skill competency. Receiving blended learning was associated with higher confidence in CIM conversations and lower avoidance of CIM conversations. Oncology physicians felt well prepared for both measures—a full conversation about CIM and a simple provision of the information leaflet—even though only providing the information leaflet about recommended websites felt impersonal. The recommended websites were used by most of the patients. Therefore, providing information about recommended CIM websites could be a suitable and easy approach for oncology physicians. However, having information about the evidence base of the use of CIM therapies in cancer care, and how to communicate about CIM, can be an option to not only meet the needs of patients asking about CIM but also reduce stress for physicians.

### Supplementary Information

**Additional file 1. **Physician–patient interaction. Qualitative results. Analysis with cluster adjustment.

## Data Availability

The datasets analyzed during the current study are available from the corresponding author upon reasonable request.

## Abbreviation

CIM: Complementary and Integrative Medicine
KOKON-KTO: Competence Network for Complementary Medicine—Consultation Training for Oncology Physicians
