# Supplementary material for: Effects of blended learning training for oncology physicians to advise their patients about complementary and integrative therapies: results from the multicenter cluster-randomized KOKON-KTO trial
Source: BMC Cancer. 2023 Sep 7;23:836. doi: 10.1186/s12885-023-11348-6 (PMC10483860; doi:10.1186/s12885-023-11348-6)
Supplement: Supplementary file 1 — Additional file 1. Physician–patient interaction. Qualitative results. Analysis with cluster adjustment. [file 12885_2023_11348_MOESM1_ESM.docx]

Supplement I: Physician–patient interaction

Table: Rating* of the consultation with the standardized patients at the end of the workshop (two raters per oncology physician, presented as the mean ± standard deviation across all physicians): Interactive and communicative competencies

|  | Gynecology  mean(±sd) (n=17) | Oncology  mean(±sd) (n=20) | Total  mean (±sd) (n=37) |
| --- | --- | --- | --- |
| *Physician:* | | | |
| let the patient speak. | 4.7 (±0.8) | 4.9 (±0.4) | 4.8 (±0.6) |
| informed the patient about the general conditions (time, content, goals) of the consultation. | 4.6 (±0.6) | 4.6 (±0.6) | 4.6 (±0.6) |
| paused in the consultation and waited to see if the patient had anything else to say. | 4.6 (±0.6) | 4.8 (±0.4) | 4.7 (±0.5) |
| repeated single words or statements that the patient said. | 4.7 (±0.8) | 4.6 (±0.7) | 4.4 (±0.8) |
| summarized, in his own words, what the patient said. | 4.2 (±0.8) | 4.8 (±0.6) | 4.5 (±0.8) |
| addressed the patient’s feelings. | 4.6 (±0.7) | 4.8 (±0.5) | 4.7 (±0.6) |
| expressed himself in simple and understandable terms. | 4.8 (±0.4) | 4.9 (±0.3) | 4.9 (±0.3) |
| explained technical terms. | 4.6 (±0.5) | 4.8 (±0.4) | 4.7 (±0.5) |
| maintained eye contact. | 5.0 (±0.0) | 4.9 (±0.3) | 5.0 (±0.2) |
| encouraged the patient to keep talking. | 4.8 (±0.5) | 4.9 (±0.3) | 4.9 (±0.4) |
| created a friendly atmosphere for the consultation. | 5.0 (±0.2) | 5.0 (±0.2) | 5.0 (±0.2) |

- *NRS from 1 fully disagree to 5 fully agree

Supplement II: Qualitative results

|  |
| --- |
|  |

**Thematic overview: Keywords from the interview data.**

|  | ***KTO* Information Leaflet** | | | ***KOKON-KTO* consultation** | | |
| --- | --- | --- | --- | --- | --- | --- |
| **Topics** | ***Potentials*** | ***Challenges*** | ***Physicians’ experiences*** | ***Potentials*** | ***Challenges*** | ***Physicians’ experiences*** |
| **Expertise** | Professional |  | Being well prepared  Feeling confident  Being able to refer to a high quality source despite the lack of own CIM knowledge | Professional → Being able to transmit expertise  High quality sources |  | Being able to address the pat’. topics and to classify them → pat. are more satisfied  Feeling confident and competent  Meeting the own quality standards |
| **Structured approach** | Addressing CIM in a structured way | Distrust towards CIM | Possibility to address CIM | Structured conversations |  | Being able to practically address CIM topics |
| **Passing information** | Additional information for pat. | Passing information passively  Pat. would wish for more and quicker information | Impersonal, dry | Actively passing information and content |  | Gaining more background knowledge  Being able to pass tools to the pat.  Being able to address concrete topics like diet, exercise and relaxation |
| **Needs** | Covers a big need of the pat. |  | Empower pat.  Provides support for pat. | Being able to take the time to address CIM | Addressing CIM with pat. is time-consuming |  |
| **Outsourcing**  **Losing control** |  | Outsourcing the CIM topic | Pat. might feel that the phys. want to outsource the CIM topic |  | “Open the Box of Pandora”  “To open a whole new can of worms” | Pat. have spiritual needs  Phys.: Losing control over content and time → stress |
| **Lack of knowledge and experience** |  | Phys.: Lack of experience → not being able to qualify | Pat. expect advises from phys.  Phys.: Lack of knowledge and experience lead to insecurity  Phys.: Lack of a professional CIM network |  |  |  |
| **Internet** | Easy access to information through internet | Requires digital Literacy |  |  |  |  |

**Representative quotes from the interviewees**

|  | *KTO* Information Leaflet | *KOKON-KTO* consultation |
| --- | --- | --- |
| Adressing CIM in a structured way | “They [patients] were already happy that the topic [CIM] was addressed at all” (A5, 17). | “The conversations were more structured after training” (A1, 55). |
| Feeling competent | “Actually,…we were well prepared, the introduction how to start was informative, insofar actually very sovereign” (A1, 5). | “I mean how you radiate a certainty and being able to say something competent about the individual topics [CIM], that satisfies the people” (A5, 47). |
| Passing information | “Well, because I had no real knowledge about the content [CIM]. So, I just handed [the leaflet] over to the people, that felt so impersonal…it felt too dry” (A5, 13-15). | “That was because you had these two points with relaxation and movement, so to speak, where you could give the patients a first tool, so to speak, they were completely satisfied with it” (A4, 31). |
| **Time efficiency and management**  **Losing control of the conversation** | “They ultimately got hold of the leaflet, were quite interested and, I think, were then looking forward to simply surfing through it” (A7, 37).” | During the longer conversation, it sometimes turns out that you…open a bit of a barrel, I must say. And then sometimes, of course, you get into a conversation and the patients of course also report what they use as a procedure or where they have had good experiences and then a Pandora's box is opened again and then yes, then you get from one thing to another and leave the medical…That increases the stress level and ultimately, you make additional work for yourself, I think so. In terms of time alone, and that is perhaps a bit of a danger…and that went better with the leaflet task. It was quite clear that we…can't talk about possibilities here, but I can at least give you the flyer, you can have a look and read by yourself, there are good sources and so on” (A2, 29). |

Supplement III: Analysis with cluster adjustment

The table shows the results of the additional statistical analyses for the relevant outcomes, adjusting for center instead of single physician. Please see the statistics section and the discussion section in the main manuscript for more details and Table 3 of the manuscript for the results of the preplanned analyses.

| Perceived consultation skill competency | | Intervention group (n=18)  mean (95% CI) | Control group (n=23)  mean (95% CI) | Group differences  mean (95% CI) | p value |
| --- | --- | --- | --- | --- | --- |
|  | Overburden | 1.4 (0.7;2.1) | 2.1 (1.4;2.8) | -0.7 (-1.7;0.3) | 0.158 |
|  | Tension | 1.3 (0.6;2.0) | 2.0 (1.3;2.7) | -0.7 (-1.7;0.3) | 0.159 |
|  | Discomfort with the consultation situation | 1.0 (0.3;1.7) | 1.8 (1.2;2.5) | -0.9 (-1.8;0.1) | 0.077 |
